# Distinct cerebrospinal fluid amyloid β peptide signatures in sporadic and *PSEN1 *A431E-associated familial Alzheimer's disease

**DOI:** 10.1186/1750-1326-5-2

**Published:** 2010-01-14

**Authors:** Erik Portelius, Ulf Andreasson, John M Ringman, Katharina Buerger, Jonny Daborg, Peder Buchhave, Oskar Hansson, Andreas Harmsen, Mikael K Gustavsson, Eric Hanse, Douglas Galasko, Harald Hampel, Kaj Blennow, Henrik Zetterberg

**Affiliations:** 1 Institute of Neuroscience and Physiology, Department of Psychiatry and Neurochemistry, The Sahlgrenska Academy at University of Gothenburg, Mölndal, Sweden; 2 UCLA Department of Neurology, Mary S Easton Center for Alzheimer Disease Research, Los Angeles, CA, USA; 3 Dementia Research Section and Memory Clinic, Alzheimer Memorial Center and Geriatric Psychiatry Branch, Department of Psychiatry, Ludwig-Maximilian University, Munich, Germany; 4 Institute of Neuroscience and Physiology, Department of Physiology, The Sahlgrenska Academy at University of Gothenburg, Gothenburg, Sweden; 5 Clinical Memory Research Unit, Department of Clinical Sciences Malmö, Lund University, Sweden; 6 University of California, San Diego, and Neurology Service, Veterans Affairs Medical Center, San Diego, CA, USA; 7 Discipline of Psychiatry, School of Medicine & Trinity College, Trinity College Institute of Neuroscience (TCIN), Laboratory of Neuroimaging & Biomarker Research, University of Dublin, Dublin, Ireland; 8 The Adelaide and Meath Hospital Incorporating the National Children's Hospital (AMiNCH), Dublin, Ireland

## Abstract

**Background:**

Alzheimer's disease (AD) is associated with deposition of amyloid β (Aβ) in the brain, which is reflected by low concentration of the Aβ1-42 peptide in the cerebrospinal fluid (CSF). There are at least 15 additional Aβ peptides in human CSF and their relative abundance pattern is thought to reflect the production and degradation of Aβ. Here, we test the hypothesis that AD is characterized by a specific CSF Aβ isoform pattern that is distinct when comparing sporadic AD (SAD) and familial AD (FAD) due to different mechanisms underlying brain amyloid pathology in the two disease groups.

**Results:**

We measured Aβ isoform concentrations in CSF from 18 patients with SAD, 7 carriers of the FAD-associated presenilin 1 (*PSEN1*) A431E mutation, 17 healthy controls and 6 patients with depression using immunoprecipitation-mass spectrometry. Low CSF levels of Aβ1-42 and high levels of Aβ1-16 distinguished SAD patients and FAD mutation carriers from healthy controls and depressed patients. SAD and FAD were characterized by similar changes in Aβ1-42 and Aβ1-16, but FAD mutation carriers exhibited very low levels of Aβ1-37, Aβ1-38 and Aβ1-39.

**Conclusion:**

SAD patients and *PSEN1 *A431E mutation carriers are characterized by aberrant CSF Aβ isoform patterns that hold clinically relevant diagnostic information. *PSEN1 *A431E mutation carriers exhibit low levels of Aβ1-37, Aβ1-38 and Aβ1-39; fragments that are normally produced by γ-secretase, suggesting that the *PSEN1 *A431E mutation modulates γ-secretase cleavage site preference in a disease-promoting manner.

## Background

Pathological hallmarks of Alzheimer's disease (AD) include synaptic and neuronal degeneration along with extracellular deposits of amyloid β protein (Aβ) in senile plaques in the cerebral cortex [1]. These changes are reflected *in vivo *by elevated tau protein concentrations and reduced levels of the aggregation prone 42 amino acid isoform of Aβ (Aβ1-42) in the cerebrospinal fluid (CSF) [2,3]. The mechanism underlying CSF Aβ1-42 reduction in AD is thought to be sequestration of the peptide in senile plaques. Accordingly, studies have found a strong correlation between low Aβ1-42 in CSF and high numbers of plaques in the neocortex and hippocampus [4], as well as high retention of Pittsburgh Compound-B (PIB) in positron emission tomography (PET) scans that directly reflect plaque pathology in the brain [5,6]. Aβ peptides are generated through proteolytic processing of the transmembrane amyloid precursor protein (APP). In the amyloidogenic pathway, APP is cleaved by two aspartyl proteases, first by β-secretase within its ectodomain and subsequently by γ-secretase within its transmembrane domain [7]. Certain forms of Aβ1-42 may act early in the disease process by disrupting synaptic plasticity mechanisms that are believed to underlie memory in the hippocampal network [8,9].

γ-Secretase is a multiprotein complex with the presenilin (PS) proteins at its enzymatic core [10]. Because of imprecise cleavage specificity, γ-secretase generates Aβ peptides of variable length at the carboxyl terminus. Mutations in the PS-encoding *PSEN1 *and *PSEN2 *genes that accelerate brain amyloid plaque pathology and cause early onset familial AD (FAD) increase the Aβ1-42/Aβ1-40 ratio in primary fibroblasts and plasma of affected individuals, in transfected cells, and in transgenic animals, but this effect is modest and not always reproducible [11,12]. To date, more than 160 distinct AD-promoting missense mutations have been identified in *PSEN1 *and three in *PSEN2*.

In addition to Aβ1-42 and Aβ1-40, there are several shorter isoforms of Aβ [13]. We recently identified a set of 18 N- and C-terminally truncated Aβ peptides in CSF using immunoprecipitation-mass spectrometry (IP-MS) [14,15]. Their relative abundance pattern distinguished AD from controls with an accuracy of 86% [16]. Here, we test the hypotheses that (i) sporadic AD patients are different from controls and patients with depression with regards to their CSF Aβ isoform pattern, (ii) SAD patients and FAD mutation carriers differ in their Aβ isoform pattern as a reflection of different mechanisms underlying brain amyloid deposition in the two disease groups, and (iii) the AD-associated Aβ1-16 fragment affects hippocampal synaptic plasticity.

## Results and Discussion

### Patient characteristics

Study participants were recruited at three specialized memory clinics, one in Munich, Germany, and two in California in the USA (UCSD and UCLA). The Munich study groups included 6 patients with SAD and 6 patients with major depression. The California study groups were comprised of 7 subjects carrying the FAD-associated *PSEN1 *A431E mutation, 12 patients with SAD and 17 healthy controls (Table 1). The A431E mutation in persons of Mexican origin represents a founder effect arising from Jalisco State [17,18]. This mutation causes an aggressive form of AD with a mean age of onset in the early 40's that is sometimes associated with spastic tetraparesis and "cotton-wool" amyloid plaques on pathology [19]. Of the 7 *PSEN1 *A431E mutation carriers, 5 were completely asymptomatic (CDR scores of 0) and had a mean age of 26 years, whereas the other two had some degree of cognitive impairment (CDR scores of 0.5 and 2, respectively, Table 2). Prior studies have demonstrated that this mutation is associated with increased levels of Aβ1-42 in the plasma of presymptomatic persons and a decreased Aβ1-42/Aβ1-40 ratio in CSF [20].

**Table 1:** Demographic characteristics of patients and controls^a^

	SAD patients (n = 18)	*PSEN1 *A431E mutation carriers (n = 7)	Healthy controls (n = 17)	Patients with depression (n = 6)
Age (years)	74 (8.9)	33 (10)	55 (17)	68 (4.9)
Gender (m/f)	10/8	3/4	7/10	0/6
MMSE scores^b^	22 (4.4)	24 (9.1)	30 (0.5)	28 (1.9)

^a^Data are presented as mean (standard deviation, SD).

^b^MMSE is Mini-Mental State Examination score.

**Table 2:** Summary of the 7 subjects with the *PSEN1 *A431E mutation

Case	APOE Genotype	Relative age^a^	CDR^b^	MMSE^c^
1	3/3	-16	0	29
2	3/3	-1	0.5	27
3	3/3	-18	0	29
4	3/3	2	2	5
5	3/3	-22	0	30
6	2/3	-19	0	28
7	2/3	-15	0	29

^a^Relative age is the number of years prior to the typical family-specific age of dementia diagnosis. Absolute age and gender are not shown to protect confidentiality with regard to subjects' identity and mutation status.

^b^CDR is the Clinical Dementia Rating scale score (0 = asymptomatic, 0.5 is questionable dementia, 1 = mild dementia, 2 = moderate dementia, 3 = severe dementia.

^c^MMSE is Mini-Mental State Examination score.

### CSF Aβ isoform patterns are distinct across groups

**Figure 1:**
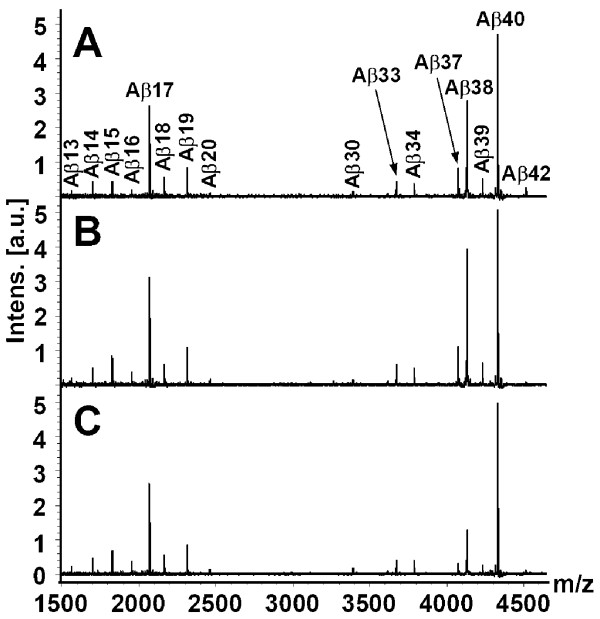
**Representative mass spectra showing C-terminally truncated Aβ peptides immunoprecipitated from cerebrospinal fluid using antibody 6E10**. (A) Representative Aβ isoform pattern in a control individual. (B) Representative Aβ isoform pattern in a patient with sporadic Alzheimer's disease. (C) Representative Aβ isoform pattern in a carrier of the FAD-associated *PSEN1 *A431E mutation.

**Figure 2:**
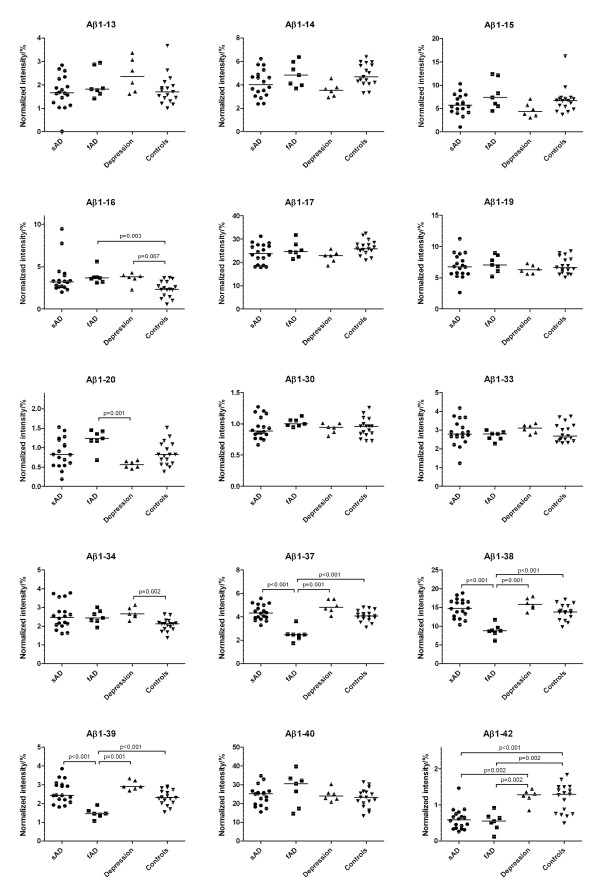
**Scatter plots of normalized Aβ fragment intensities derived from IP-MS in the different disease and control groups**. Thin horizontal lines indicate medians. Significant differences are indicated.

**Figure 3:**
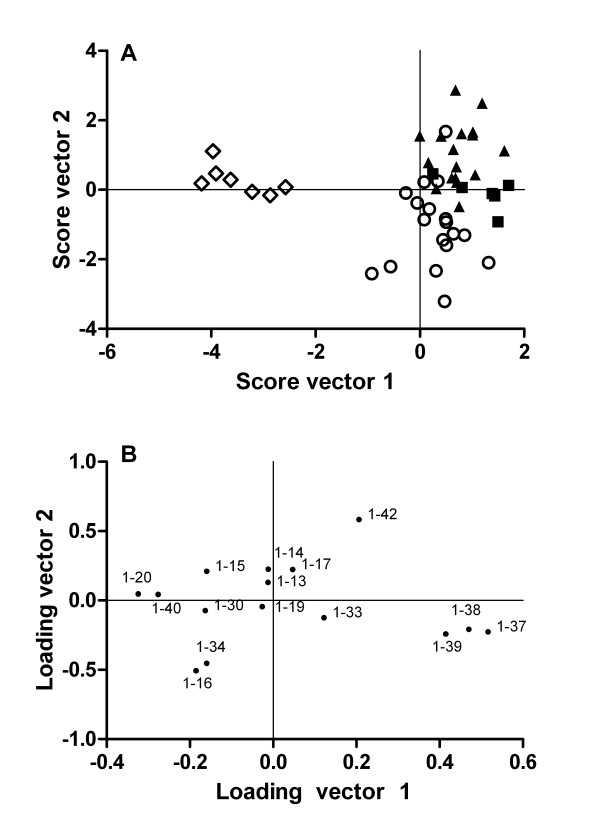
**Distinct Aβ isoform patterns in FAD compared to SAD and non-AD**. The figure shows OPLS-DA on the three classes FAD, SAD, and non-AD (controls and depression) using the data shown in figure 2. (A) Score plot for FAD (open diamonds), SAD (open circles), depression (solid squares), and controls (solid triangles). (B) Loading and plot for the Aβ fragments.

**Figure 4:**
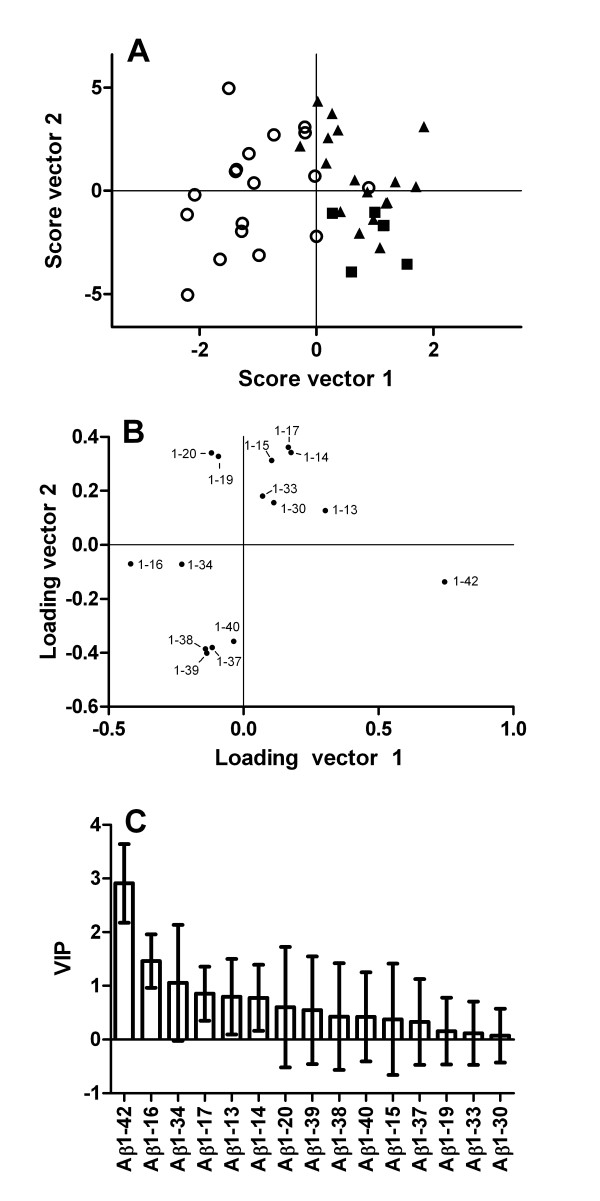
**The Aβ isoform pattern separates SAD from controls and depressed patients**. The figure shows OPLS-DA on the two classes SAD and non-AD (controls and depression) using the data shown in figure 2. (A) Score plot for SAD (open circles), depression (solid squares) and controls (solid triangles). (B) Loading and (C) variable importance on projection (VIP) plots for the Aβ fragments. The error bars in (C) represent a 95% confidence interval.

Representative CSF Aβ isoform mass spectra for SAD patients, FAD mutation carriers and controls are shown in Figure 1. Normalized CSF Aβ isoform intensities (Figure 2) were compared across the three groups using multivariate discriminant analysis (Figure 3). FAD patients were clearly separated from SAD and non-AD (controls and depression), and the latter two groups were also segregated from each other, although to a lesser extent. In order to ease the interpretation, subsequent pairwise discriminant analysis were performed for SAD patients vs. non-AD and SAD vs. FAD. Low levels of Aβ1-42 and high levels of Aβ1-16 were the main contributors for the separation of SAD from non-AD (Figure 4). Aβ1-34, Aβ1-17, Aβ1-13 and Aβ1-14 contributed weakly to the separation. Low CSF Aβ1-42 is a well-replicated finding in AD [21]. However, elevated Aβ1-16 in AD is less well known. The data presented herein, along with earlier results from independent data sets [16], show that SAD patients tend to express high levels of Aβ1-16 in their CSF at the group level, which also seems to hold true for *PSEN1 *A431E mutation carriers (Figure 2). Two SAD patients had very high Aβ1-16 levels (Figure 2). These patients, one male and one female, were 77 and 79 years old and did not differ from other SAD patients with regards to cognitive scores or Aβ1-42 concentrations. The reason for their very high Aβ1-16 levels is at present unknown.

Recent cell culture experiments using different secretase inhibitors suggest that Aβ1-16 is derived from concerted cleavages of APP by β - and α-secretase, thus reflecting a third metabolic pathway for APP [22]. Curiously, depressed patients in this study also had higher Aβ1-16 levels than the healthy controls (Figure 2). Pending confirmation in independent and larger patient materials, this result may provide clues regarding altered APP metabolism in depression. There were no other Aβ-related changes in common in depression and SAD vs. controls.

### FAD mutation carriers express low levels of Aβ1-37, Aβ1-38 and Aβ1-39 in CSF

**Figure 5:**
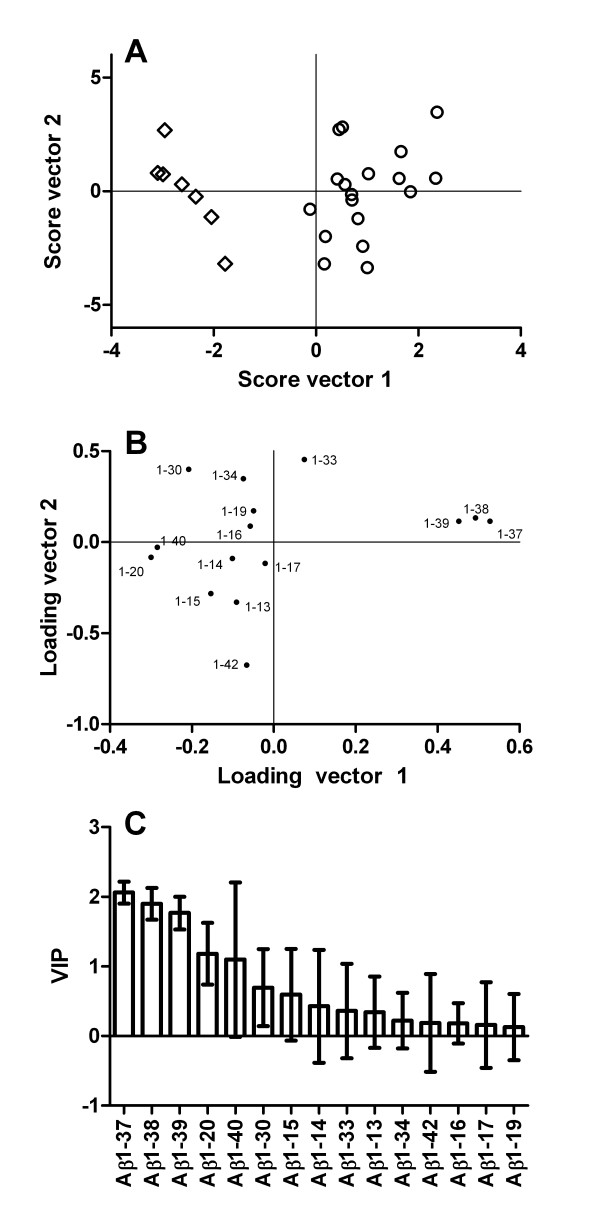
**Distinct Aβ isoform patterns in FAD and SAD**. The figure shows OPLS-DA on the two classes SAD and FAD using the data shown in figure 2. (A) Score plot for FAD (open diamonds) and SAD (open circles). (B) Loading and (C) variable importance on projection (VIP) plots for the Aβ fragments. The error bars in (C) represent a 95% confidence interval.

The reason for the distinct subgrouping of SAD patients and FAD mutation carriers in Figure 3A was analyzed in detail by comparing their CSF Aβ isoform patterns specifically (Figure 5A). Both disease groups were characterized by similar levels of Aβ1-42 and Aβ1-40, implying similar degrees of amyloid pathology in their brains [4,5]. However, FAD mutation carriers had very low concentrations of Aβ1-37, Aβ1-38 and Aβ1-39 and high Aβ1-20 compared with SAD patients (Figure 2). These deviations separated the two groups completely (Figure 5B and 5C). Similar Aβ1-37, Aβ1-38 and Aβ1-39 changes have been seen in media from cell lines expressing the *PSEN1 *Δ9 or L166P mutation, or the *PSEN2 *N141I mutation [11]. The Aβ1-37, Aβ1-38 and Aβ1-39 isoforms are normally produced by γ-secretase, suggesting that certain *PSEN1 *and *PSEN2 *mutations may modulate γ-secretase function by inhibiting cleavage at Gly37, Gly38 and Val39, without affecting the production of Aβ1-42 and Aβ1-40 significantly. It is tempting to speculate that Aβ1-37, Aβ1-38 and Aβ1-39 may inhibit Aβ1-42 oligomerization by forming less aggregation-prone heterocomplexes with Aβ1-42. Such a protective effect has recently been described for Aβ1-40 [23,24]. The key AD-promoting effect of *PSEN1 *A431E, and possibly several other FAD-associated *PSEN *mutations, may thus be a tweaked γ-secretase cleavage site preference that results in loss of C-terminally truncated Aβ species. Modulating γ-secretase function to boost cleavages at Gly37, Gly38 and Val39 would in that case be a novel approach to prevent AD-associated Aβ aggregation. However, prior to such a claim, the hypothesis that Aβ1-37, Aβ1-38 and Aβ1-39 indeed inhibit Aβ1-42 oligomerization and toxicity must be tested in additional studies.

### AD-associated Aβ1-16 does not inhibit long-term potentiation

**Figure 6:**
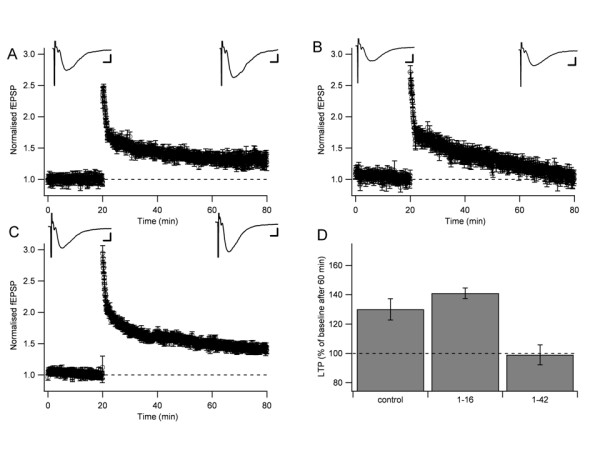
**Aβ1-16 does not inhibit hippocampal long-term potentiation (LTP)**. (A) Control LTP elicited by three trains (20 impulses, 50 Hz) separated by 5 seconds. Data points are normalized field EPSP initial slope measurements ± standard errors of the mean. Average (n = 20) field EPSPs before and 60 minutes after the induction of LTP are shown on top. (B) LTP elicited in the presence of oligomerized Aβ1-42 (positive control). (C) LTP elicited in the presence of Aβ1-16. Scale bars represent 5 ms and 0.1 mV. (D) Comparison between LTP 60 minutes after the induction in control, in Aβ1-16 and in Aβ1-42.

One of the hallmark synaptotoxic effects of Aβ1-42 is the inhibition of long-term potentiation (LTP) [25]. Results presented here, together with earlier data from our group [16], showing elevated CSF levels of Aβ1-16 in AD, prompted us to test whether the Aβ1-16 peptide *per se *inhibits LTP. To that end, we exposed acute rat hippocampal slices to Aβ1-16 and elicited LTP at the glutamatergic synapses in the CA1 region. Under our control conditions, a strong LTP-inducing protocol (three times 20 impulses at 50 Hz during blockade of GABA_A _receptors) resulted in LTP that amounted to 130 ± 7.3% (presynaptic volley = 97 ± 3.5%, n = 7) 60 minutes after the induction (Figure 6A, D). As a positive control, we exposed the slice to Aβ1-42 oligomers (prepared from 1 μM monomeric Aβ1-42, see Methods) for 30-60 minutes before the induction of LTP. Under these conditions the LTP was 103 ± 5.9% (presynaptic volley = 90.5 ± 1.6%, n = 6), which was significantly smaller than control (*P *= 0.017) (Figure 6B, D). To test whether Aβ1-16 affects the generation of LTP, we exposed the slice to Aβ1-16 (1 μg/L) for 60 minutes before the induction. The absolute endogenous concentration of Aβ1-16 in human CSF is 10-50 ng/L [15], but the synaptic concentration is not known. Therefore, to ascertain a not too low synaptic concentration of Aβ1-16, we used a concentration 20-100 times the absolute endogenous concentration of Aβ1-16 in human CSF. In the presence of Aβ1-16, LTP was 141 ± 3.6% (presynaptic volley = 100 ± 2.5%, n = 12) (Figure 6C, D), which is not significantly different from the control (*P *= 0.12). Hence, we conclude that Aβ1-16 does not inhibit LTP at hippocampal CA3-CA1 synapses.

### Limitations

Although the findings of this study are intriguing, there are some limitations that should be mentioned.

First, the study is small and the important findings, i.e., the increased levels of Aβ1-16 in AD and depression and the reduced levels of Aβ1-37, Aβ1-38 and Aβ1-39 in *PSEN1 *A431E-caused FAD, are in need of replication.

Second, CSF samples were obtained at different centers. However, no center effects on Aβ isoform levels were detected. Further, when levels of the various Aβ peptides were compared between the 7 FAD mutation carriers and their 3 similarly aged non-mutation carrying kin from whom CSF was obtained at the same center, levels of Aβ1-37, Aβ1-38 and Aβ1-39 but not of other Aβ peptides were significantly lower (*P *≤ 0.003) and non-overlapping. In fact, differences in these levels were greater than that of Aβ1-42, which was non-significant in this small subpopulation. The finding of decreased levels of Aβ1-37, Aβ1-38 and Aβ1-39 in the CSF of persons with the A431E *PSEN1 *mutation therefore appears to be a robust finding. How this might be related to the cotton wool plaque pathology that has been demonstrated to consist of increased amounts of N-terminally truncated forms of Aβ42 in persons with other *PSEN1 *mutations [26] is unclear.

Third, the age distribution differed between the different groups. Of all the Aβ isoforms, in all study groups, only Aβ1-34 in the controls correlated with age (r_s _= -0.61, *P *= 0.01). However, Aβ1-34 is higher in the AD group compared with the controls, which is opposite to what would have been expected if the difference were due to an age effect. This makes age an unlikely confounder.

## Conclusions

The findings presented here show that (i) SAD patients differ from cognitively normal individuals and depressed patients with regards to their CSF Aβ isoform pattern and (ii) carriers of the FAD-associated *PSEN1 *A431E mutation have low CSF levels of C-terminally truncated Aβ peptides shorter than Aβ1-40, suggesting a loss of function effect that leads to a relative abundance of aggregation-prone Aβ1-42. The influence of Aβ1-37, Aβ1-38 and Aβ1-39 reductions on Aβ1-42 oligomerization and toxicity needs to be examined in experimental studies. CSF Aβ1-16 may be a positive biomarker for AD but its specificity against depression must be tested further.

## Methods

### Study participants

Patients with SAD and major depression were diagnosed according to DSM-IIIR criteria [27]. SAD patients fulfilled the criteria of probable AD defined by NINCDS-ADRDA (National Institute of Neurological and Communicative Disorders - Stroke/Alzheimer's Disease and Related Disorders Association) [28]. The seven persons carrying the A431E mutation in PSEN1 [17] were participants in a study of symptomatic persons affected by (n = 2), and asymptomatic persons at-risk for (n = 5) FAD being conducted at UCLA. Subjects seen at UCLA underwent a comprehensive clinical evaluation by investigators blind to their genetic status that included the Clinical Dementia Rating scale [29]. Three of the 17 controls were non-mutation carrying family members also enrolled in this study. The healthy controls were mainly recruited from senior citizen organizations and through information meetings on dementia. A few controls were spouses of subjects in the study. Inclusion criteria for controls were that they should be physically and mentally healthy and not experiencing or exhibiting any cognitive impairment. All controls were thoroughly interviewed about their somatic and mental health by researchers before inclusion in the study. Mini-mental state examination (MMSE) was used as a global measure of cognitive functioning [30]. The study was approved by the ethics committees of Ludwig-Maximilian University, Germany, and UCLA and UCSD, USA.

### CSF sampling and biochemical analyses

CSF samples were collected in the morning by lumbar puncture (LP) through the L3/L4 or L4/L5 interspace. CSF was collected in polypropylene tubes in 500 μL aliquots that were centrifuged, frozen and stored at -80°C pending biochemical analyses, without being thawed and re-frozen. The immunoprecipitation and mass spectrometric analysis were conducted as described before [15]. Briefly, 8 μg of the monoclonal antibody 6E10 (epitope 4-9, Signet Laboratories Inc., Dedham, USA) was used together with magnetic Dynabeads (Sheep anti-mouse IgG) for immunoprecipitating C-terminally truncated Aβ peptides from 1 mL CSF. The samples were analyzed by matrix-assisted laser desorption/ionization time-of-flight mass spectrometry (MALDI-TOFMS, Autoflex, Bruker Daltonics, Bremen, Germany) operating in reflector mode. An in house MATLAB^® ^program (Mathworks Inc. Natick, MA, USA) was used for integration of the peaks for each spectrum and the integration limits were from-2 to +5 m/z relative to the monoisotopic peak. Prior to the statistical analysis the peak areas were normalized to the sum of the integrated peaks.

### Electrophysiology

Electrophysiological experiments were performed on hippocampal slices from 35-60 day-old male Wistar rats. The rats were anesthetized with isoflurane (Abbott) prior to decapitation. The brain was removed and placed in an ice-cold solution containing (in mM): 140 cholineCl, 2.5 KCl, 0.5 CaCl_2_, 7 MgCl_2_, 25 NaHCO_3_, 1.25 NaH_2_PO_4_, 1.3 ascorbic acid and 7 dextrose. Transverse hippocampal slices (400 μm thick) were cut with a vibratome (HM 650 V Microm, Germany) in the same ice-cold solution and were subsequently stored in artificial cerebrospinal fluid (ACSF) containing (in mM): 124 NaCl, 3 KCl, 2 CaCl_2_, 4 MgCl_2_, 26 NaHCO_3_, 1.25 NaH_2_PO_4_, 0.5 ascorbic acid, 3 myo-inositol, 4 D, L-lactic acid, and 10 D-glucose. After at least one hour of storage at 25°C, a single slice was transferred to a recording chamber where it was kept submerged in a constant flow (~2 ml min^-1^) at 30-32°C. The perfusion fluid contained (in mM) 124 NaCl, 3 KCl, 4 CaCl_2_, 4 MgCl_2_, 26 NaHCO_3_, 1.25 NaH_2_PO_4_, and 10 D-glucose. Picrotoxin (100 μM, Sigma-Aldrich Stockholm, Sweden) was always present in the perfusion fluid to block GABA_A _receptor-mediated activity. All solutions were continuously bubbled with 95% O_2 _and 5% CO_2 _(pH ~7.4). The higher than normal Ca^2+ ^and Mg^2+ ^concentrations were used to inhibit spontaneous network activity. ACSF was spiked with synthetic Aβ1-16 (Bachem, Weil am Rhein, Germany) in water solution to a final concentration of 1 μg/L. Aβ1-42 oligomers were prepared according to a standard protocol [31]. Briefly, 1 μM Aβ1-42 was dissolved in 1,1,1,3,3,3-hexofluoro-2-propanol (HFIP) on ice and incubated for 90 minutes in room temperature. HFIP was removed using speedvac and the remaining Aβ1-42 peptide film was stored at -80°C. The film was dissolved in DMSO to 5 mM, sonicated, further diluted in PBS containing 0.2% SDS to 400 μM and incubated for six hours at 37°C. Water was added to a concentration of 100 μM and this solution was incubated for 18 hours at 37°C. Finally, the solution was centrifuged at 3000 g for 20 minutes and stored for no more than three days at 4°C.

Electrical stimulation of Schaffer collateral/commissural axons and recordings of synaptic responses were carried out in the stratum radiatum of the CA1 region. Stimuli consisted of biphasic constant current pulses (15-80 μA, 200 μS, STG 1002 Multi Channel Systems MCS Gmbh, Reutlingen, Germany) delivered through tungsten wires (resistance ~0.1 MΩ). The synaptic input was activated every 5 s. Field excitatory postsynaptic potentials (EPSPs) were recorded with a glass micropipette (1 M NaCl, resistance ~4 MΩ). Field EPSPs were sampled at 10 kHz with an EPC-9 amplifier (HEKA Elektronik, Lambrecht, Germany) and filtered at 1 kHz. Evoked responses were analyzed off-line using custom-made IGOR Pro (WaveMetrics, Lake Oswego, OR) software. Field EPSP magnitude was estimated by linear regression over the first 0.8 ms of the initial slope. The presynaptic volley was measured as the slope of the initial positive-negative deflection, and it was not allowed to change by more than 15% during the experiment.

### Statistical analyses

Multivariate discriminant analysis (DA) was performed using the orthogonal projection to latent structure (OPLS) algorithm [32] implemented in the software SIMCA P+ v. 12 (Umetrics, Umeå, Sweden). In general, OPLS-DA finds the direction (score vector) in the multidimensional orthogonal space created by the different measured variables that best separate the predefined classes. To visualize the result from an OPLS-DA, the observations are projected onto a plane spanned by the score vectors. The contribution of the different variables to the score vectors is presented in a loading plot. A vector from the origin to a variable in the loading plot points in the direction that an observation in the score plot will be displaced if the value of the variable is increased. Also, the extent of the displacement is proportional to the magnitude of the vector [33]. Comparisons between groups with regards to individual, normalized Aβ isoform intensities were performed using nonparametric Kruskal-Wallis test, followed by the Mann-Whitney test. P-values for the Mann-Whitney test were reported given that (i) the p-value for the Kruskal-Wallis was below 0.05 after Bonferroni correction (15 tests) and (ii) the difference was significant at p < 0.05 using Dunn's post hoc test for multiple comparisons. Electrophysiological data were evaluated using Student's t-test.

## Competing interests

The authors declare that they have no competing interests.

## Authors' contributions

EP participated in the design of the study and carried out mass spectrometric analyses. UA participated in the design of the study and performed statistical analyses. JMR characterized patients and contributed samples. KBu characterized patients and contributed samples. JD performed electrophysiological experiments. PB and OH participated in the design of the study. AH performed electrophysiological experiments. MKG carried out mass spectrometric analyses. EH performed electrophysiological experiments. DG and HH characterized patients and contributed samples. KBl participated in the design of the study and its coordination. HZ participated in the design of the study and its coordination, and drafted the manuscript. All authors interpreted the data, revised the manuscript for important intellectual content and read and approved the final manuscript version.
